# Catheter ablation of arrhythmias: 15 years of development: data from the Swedish Catheter Ablation Registry

**DOI:** 10.1093/ehjopen/oeaf142

**Published:** 2025-10-23

**Authors:** Christine Mannewald, Pyotr G Platonov, Espen Fengsrud, Niklas Höglund, Lars O Karlsson, Stefan Lönnerholm, Jonas Schwieler, Michael Ringborn, Rúna Landén, Fariborz Tabrizi, Jari Tapanainen, Frieder Braunschweig, Fredrik Holmqvist

**Affiliations:** Department of Cardiology, Clinical Sciences, Lund University, SE-221 85 Lund, Sweden; Department of Cardiology, Clinical Sciences, Lund University, SE-221 85 Lund, Sweden; Department of Cardiology, University Hospital Örebro, SE-701 85 Örebro, Sweden; Department of Cardiology, Umeå University Hospital, SE-901 89 Umeå, Sweden; Department of Cardiology, Linköping University Hospital, SE-581 85 Linköping, Sweden; Department of Cardiology, Uppsala University Hospital, SE-751 85 Uppsala, Sweden; Department of Cardiology, Karolinska University Hospital, SE-171 76 Stockholm, Sweden; Thoracic Center Blekinge County Hospital, SE-371 85 Karlskrona, Sweden; Institution for Medicine, Sahlgrenska Academy, SE-413 45 Gothenburg, Sweden; Department of Clinical Sciences, Karolinska Institute, South Hospital, Arrhythmia Center Stockholm, SE-118 83 Stockholm, Sweden; Department of Cardiology, Karolinska Institute, Danderyd Hospital, Division of Cardiovascular Medicine, SE-182 88 Stockholm, Sweden; Department of Cardiology, Karolinska University Hospital, SE-171 76 Stockholm, Sweden; Department of Cardiology, Clinical Sciences, Lund University, SE-221 85 Lund, Sweden

**Keywords:** Arrhythmias, Catheter ablation, Atrial fibrillation

## Abstract

**Aims:**

The number of patients undergoing catheter ablation is continuously growing, and techniques are improving. However, studies reporting contemporary data on catheter ablations from large real-world populations are scarce. This study aims to report characteristics and outcomes of catheter ablation from 2006 to 2020, using a nationwide registry with virtually complete coverage.

**Methods and results:**

From the Swedish Catheter Ablation Registry, patients >18 years of age undergoing catheter ablation from 2006 to 2020 were included. Periprocedural data and baseline characteristics were recorded retrospectively. A total of 61 243 procedures were included. There was an overall increase in the number of catheter ablations performed. From 2006, the number of atrial fibrillation (AF) ablations performed increased from 352 procedures in 2006 to 2609 procedures in 2020. Decreased procedural times were seen in catheter ablation of accessory pathway/Wolff–Parkinson–White syndrome, atrial tachycardia (AT), atrioventricular nodal reentry tachycardia, cavotricuspid isthmus (CTI), AF, and atrioventricular junction. Between the time periods 2006–15 and 2016–20, median procedural time in AF ablations decreased from 180 to 140 min (*P* < 0.001). There was a decreased trend in fluoroscopy time and median dose area product for all ablation procedures (*P* < 0.001). For AT, CTI, and AF, the cumulative probability of requiring a repeat ablation was significantly lower for procedures performed after January 2016 (*P* < 0.001).

**Conclusion:**

With a yearly increase in the number of ablations performed, there was a reduction in the need for repeat ablations for AF, AT, and CTI, along with reduced procedural times and lower fluoroscopy levels.

## Graphical Abstract

**Graphical Abstract:**
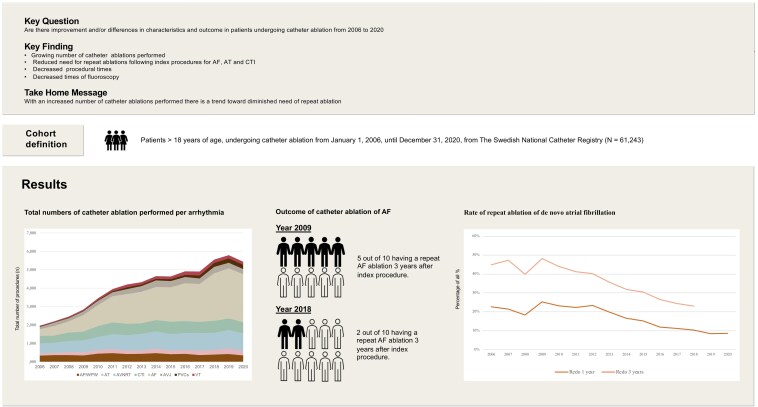

## Introduction

After four decades of development, catheter ablation is established as a safe and effective treatment option for cardiac arrhythmias, with a high success rate for most procedures,^1–3^ and is currently considered a first-line treatment for a range of arrhythmias.^3^ For atrial fibrillation (AF), catheter ablation has been shown to be the most efficient method for achieving AF burden reduction and maintenance of sinus rhythm.^4^ Given its expanding indications, higher demand, and technical improvements, the number of catheter ablation procedures performed each year is increasing. In addition, catheter ablation is now performed in more advanced arrhythmias, such as complex atrial arrhythmias and ventricular tachycardia (VT).^5^ Although the role of fluoroscopy has diminished with the introduction of 3D-mapping systems, the use of fluoroscopy is still necessary in most catheter ablation procedures.

Studies on outcomes and periprocedural data from large, representative patient populations reflective of patients currently treated in routine clinical practice are scarce. Existing publications are relatively small in scale, or they relate to investigating specific outcomes or specific arrhythmias.^6–8^ The Swedish Catheter Ablation Registry is a nationwide registry encompassing virtually all ablations performed in Sweden since its initiation in 2005. Due to advancements in techniques, which have facilitated potentially faster procedures and reduced fluoroscopy use, this study sought to report the characteristics and outcomes of catheter ablation from 2006 to 2020, and to compare findings between patients being ablated from 2006 to 2015, with patients ablated from 2016 to 2020.

## Methods

### Study population

Patients of at least 18 years of age, who underwent catheter ablation at 1 of the 11 ablation centres (7 university hospitals, 3 community hospitals, and 1 private institution) in Sweden between 1 January 2006 and 31 December 2020 were included. Follow-up data (repeat ablation) were recorded until 31 December 2021.

### Data collection

Data collection was performed using the Swedish Catheter Ablation Registry. The Registry was initiated in 2005, and since 2006, every centre in Sweden performing catheter ablation of cardiac arrhythmias reports to the Registry. The Registry has been described previously and has been utilized to analyse trends and outcomes in a prior publication (reporting data from 2006 until 2015).^9^ To compare this previously reported group, a cohort of a similar size was chosen. From 2016 until 2020, a comparable number of patients had undergone ablations, and therefore constitute the second group. Patient consent was obtained prior to the procedure, with an opt-out alternative. Baseline characteristics, together with procedural characteristics, are prospectively recorded. Coverage and data completeness are high throughout the studied time (see Supplementary material). Acute success was first reported in 2008 for cavotricuspid isthmus (CTI) ablation and in 2009 for AF, VT, and premature ventricular contractions (PVCs). First-time and repeat ablation procedures for various types of arrhythmias were recorded for each patient as identified by their unique Swedish patient identification number. Patients who underwent multiple ablation procedures for different arrhythmias were counted separately for each type of ablation procedure performed. If ablation of more than one arrhythmia substrate [e.g. atrioventricular nodal reentry tachycardia (AVNRT) and CTI] was performed in the same session, the patient contributed to data for each arrhythmia substrate in the Registry. The exception was when AF and CTI ablation were simultaneously performed, in which case only AF ablation was reported. When analysing the risk of repeat ablation, only first-time ablation procedures that were deemed acutely successful were considered. Ablation for AF was performed with pulmonary vein isolation in *de novo* ablations. In the event of arrhythmia recurrence, whether AF or macro-reentrant atrial tachycardia (AT), the procedure was registered as a repeat ablation for AF in the Registry. Atrial fibrillation subtypes were not reported until 2016. Atrial fibrillation subtypes were divided into paroxysmal <7 days, persistent <1 year, and longstanding persistent >1 year. The presence of comorbidities (e.g. heart failure) was not reported until 2016 and is not complete in registration, and is therefore not included in the study. The study was approved by the Swedish Ethical Review Authority and was conducted in accordance with the Declaration of Helsinki.

### Statistics

Continuous variables are presented as mean ± standard deviation or median with interquartile range when appropriate. Comparisons were made using the Student’s *t*-test or the Mann–Whitney *U* test according to their distributions. Categorical values are reported as percentages and compared using the χ^2^ test. Comparison between different years of catheter ablation was performed using the Mantel–Haenszel test (categorical data). In the cumulative comparison, patients were censored if lost to follow-up or if death occurred. Comparisons between different years were made using the log-rank test. All tests performed were two-sided and considered significant if *P* < 0.05. All analysis was performed using IBM SPSS Statistics (IBM SPSS Statistics for Mac, version 29.0.0.0, Armonk, NY, USA).

## Results

### Study population

A total of 61 243 procedures were included in the analysis, of which 34 636 procedures were performed between 1 January 2006 and 31 December 2015, and 26 607 procedures were performed between 1 January 2016 and 31 December 2020. Age, gender, concomitant disease, and peri-operative data are presented per arrhythmia type in *Table 1*. Female dominance was seen in ablation of AVNRT, atrioventricular junction (AVJ), and PVCs. From 2006 to 2020, there was a trend towards an increasing median age in all arrhythmias, except for ablation of accessory pathway/Wolff–Parkinson–White syndrome (AP/WPW). Patients undergoing ablation for AP/WPW were the youngest patients throughout the studied period, with a median age of 41.

**Table 1:** Baseline characteristics

	AP/WPW		AT		AVNRT		CTI	
	2006–15	2016–20	2006–15	2016–20	2006–15	2016–20	2006–15	2016–20
	*n* = 4091	*n* = 1980	*n* = 1810	*n* = 1252	*n* = 7384	*n* = 4729	*n* = 5486	*n* = 3161
Median age (IQR)—years	41 (27–54)	41 (27–54)	59 (44–68)	61 (45–71)*	55 (42–65)	56 (44–67)*	64 (55–70)	66 (58–73)*
Male gender—%	62	62	50	48	40	41.8*	80	79
Median weight (IQR)—kg	78 (67–89)	79 (68–90)*	80 (67–90)	78 (67–90)	76 (66–88)	78 (67–90)*	85 (75–95)	85 (75–97)
Median BMI (IQR)	25.8 (23.4–28.0)	25.1 (22.8–28.4)	27.2 (24.2–30.2)	25.9 (23.0–29.4)	25.9 (23.5–29.4)	25.7 (23.1–29.4)	26.0 (24.7–29.8)	26,6 (24,2–29,8)
Heart disease—%								
Ischaemic heart disease	4.0	2.5*	11	8.1*	8.1	5.1*	19	14.8*
Dilated cardiomyopathy	1.7	0.7	7.2	5	1.6	1	12	8.1*
Hypertrophic cardiomyopathy	0.6	0.4	0.9	1.3	0.4	0.2	1.5	1.3
ARVC	0	0	0.0	0.4*	0.0	0.1	0.1	0.1
Median procedure time (IQR)—min	120 (90–160)	115 (87–153)*	175 (130–220)	160 (120–210)*	100 (80–129)	95 (75–120)*	105 (80–140)	90 (70–120)*
Median fluoroscopy time (IQR)—min	14 (8–24)	10 (6–17)*	16 (10–27)	10 (6–16)*	8 (5–14)	6 (4–10)*	14 (8–24)	9 (5–16)*
Median radiation dose (IQR)—cGycm^2^	970 (400–2200)	409 (196–80)*	977 (408–2244)	355 (166–778)*	462 (200–1000)	220 (101–475)*	1043 (470–2200)	400 (192–860)*
RF ablation—%	94	94	87	94	66	68	76	93
Median RF time (IQR)—s	152 (77–315)	192 (100–370)*	480 (236–992)	558 (270–1052)*	133 (75–256)	158 (88–285)*	720 (424–1208)	733 (453–1200)*
Median RF energy (IQR)—kJ	5160 (2758–10 279)	6221 (3369–11 927)*	15 921 (7643–30 538)	15 587 (7320–28 660)	4011 (2333–7581)	4861 (2813–8741)*	24 178 (14 248–40 423)	23 945 (15 040–38 583)
Cryo-ablation—%	8.8	10	16	10	36	36	27	8
Median cryo time (IQR)—s	480 (256–796)	540 (298–842)	950 (514–1680)	725 (480–1260)*	720 (480–1130)	803 (540–1201)*	2388 (1680–3346)	1850 (1214–2800)*
Acute success—%	91	92	79	82	97	96*	95	96*

	AF		AVJ		PVCs		VT	
	2006–15	2016–20	2006–15	2016–20	2006–15	2016–20	2006–15	2016–20
	*n* = 11 907	*n* = 12 002	*n* = 2412	*n* = 1576	*n* = 578	*n* = 955	*n* = 968	*n* = 926
Median age (IQR)—years	61 (54–67)	63 (56–70)*	74 (68–80)	76 (71–81)*	50 (38–62)	53 (42–66)*	61 (48–70)	62 (43–66)*
Male gender—%	72	71*	47	46	44	48	74	81*
Median weight (IQR)—kg	87 (77–97)	87 (77–97)	78 (68–91)	80 (69–92)	80 (69–90)	78 (69–89)	83 (73–93)	85 (76–96)*
Median BMI (IQR)	26.6 (24.3–29.3)	26.9 (24.6–29.7)	24.3 (22.9–25.2)	26.8 (23.9–30.9)*	27.9 (20.8–35.0)	25.6 (22.9–29.0)	21.6 (21.6–21.6)	26.9 (24.5–30.2)
Heart disease—%								
Ischaemic heart disease	8.2	7.3*		25.2		8.9		46.2
Dilated cardiomyopathy	3.7	4.7*		23.7		13.1		25.8
Hypertrophic cardiomyopathy	1.9	1.3*		1.8		0.8		1.7
ARVC	0	0.1		0.3		0.2		4.6
Median procedure time (IQR)—min	180 (140–220)	144 (112–180)*	60 (44–75)	52 (40–70)*	153 (120–186)	160 (123–210)*	177 (139–225)	210 (154–285)*
Median fluoroscopy time (IQR)—min	21 (13–34)	12 (8–17)*	5 (3–10)	4 (2–8)*	11 (6–20)	10 (5–17)*	16 (10–29)	16 (9–25)*
Median radiation dose (IQR)—cGycm^2^	1882 (970–3691)	623 (340–1130)*	308 (121–800)	174 (80–400)*	568 (200–805)	330 (142–728)*	1700 (604–3900)	882 (400–1814)*
RF ablation—%	88	80	99	99.9	99	99	98	100
Median RF time (IQR)—s	2392 (1571–3491)	2012 (1328–2739)*	150 (79–336)	156 (90–345)	451 (270–805)	506 (291–919)	746 (400–1550)	1200 (555–2247)*
Median RF energy (IQR)—kJ	65 865 (44 327–96 906)	62 267 (40 522–83 717)*	3548 (1900–8063)	4975 (2699–9873)*	14 988 (7100–31 137)	17 092 (9486–29 789)	37 897 (16 894–66 382)	48 280 (19 240–87 773)*
Cryo-ablation—%	13	21	0.5	0.1	3.8	1	1.1	0
Median cryo time (IQR)—s	2160 (1680–3067)	1030 (891–1325)*	1387 (543–2298)		730 (251–1578)	850 (322–1170)	1200 (480–1898)	
Acute success—%	97	98*	97	98	82	86	86	85

Fields with <60% are not reported.

AP/WPW, accessory pathway/Wolff–Parkinson–White syndrome; AT, atrial tachycardia; AVNRT, atrioventricular nodal reentry tachycardia; CTI, cavotricuspid isthmus; AF, atrial fibrillation; AVJ, atrioventricular junction; PVCs, premature ventricular contractions; VT, ventricular tachycardia; ARVC, arrhythmogenic right ventricular cardiomyopathy; RF, radiofrequency; BMI, body mass index; IQR, interquartile range.

Analyses performed using Student’s *t*-test, or χ^2^ when deemed necessary. *P < 0.05.

### Procedural data

Procedural times were decreasing for all arrhythmias, with the exception of ablation of PVCs and VT, which presented an increase from 153 to 160 and 177 to 210 min, respectively (*P* = 0.003 for PVCs; *P* < 0.001 for VT). The largest decrease was seen in ablation of AF with a median procedural time of 180 min during the period 2006–15, vs. 144 min during the period 2016–20 (*P* < 0.001), see *Table 1*. Radiofrequency (RF) was the predominant technique used in all arrhythmias. Acute success remained >90% for AP/WPW, AVNRT, CTI, AF, and AVJ during both studied periods.

**Figure 1:**
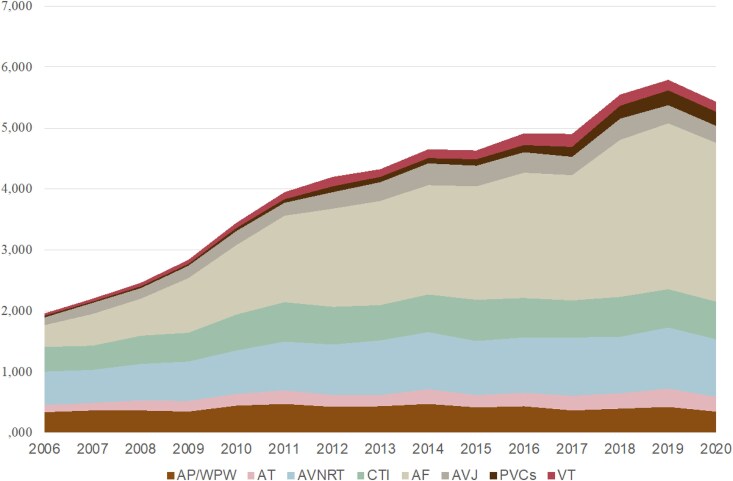
Numbers of ablations performed per year. Cumulative distribution among arrhythmias, reported per year. AP/WPW, accessory pathway/Wolff–Parkinson–White; AT, atrial tachycardia; AVNRT, atrioventricular nodal reentry tachycardia; CTI, cavotricuspid isthmus; AF, atrial fibrillation; AVJ, atrioventricular junction; PVCs, premature ventricular contractions; VT, ventricular tachycardia.

Ablations performed for AF, VT, and PVCs showed an overall increase from 2006 to 2015, compared with the period of 2016–20. Atrial fibrillation ablation was the most performed procedure in both time periods. During the 2006–15 period, a total of 11 907 (34.4%, 11 907/34 636) ablations of AF were performed. During the 2016–20 period, the total number of AF ablations was 12 002 (45.1%, 12 002/26 607). For distribution among all arrhythmias, see *Figure 1*.

### Fluoroscopy utilization

**Figure 2:**
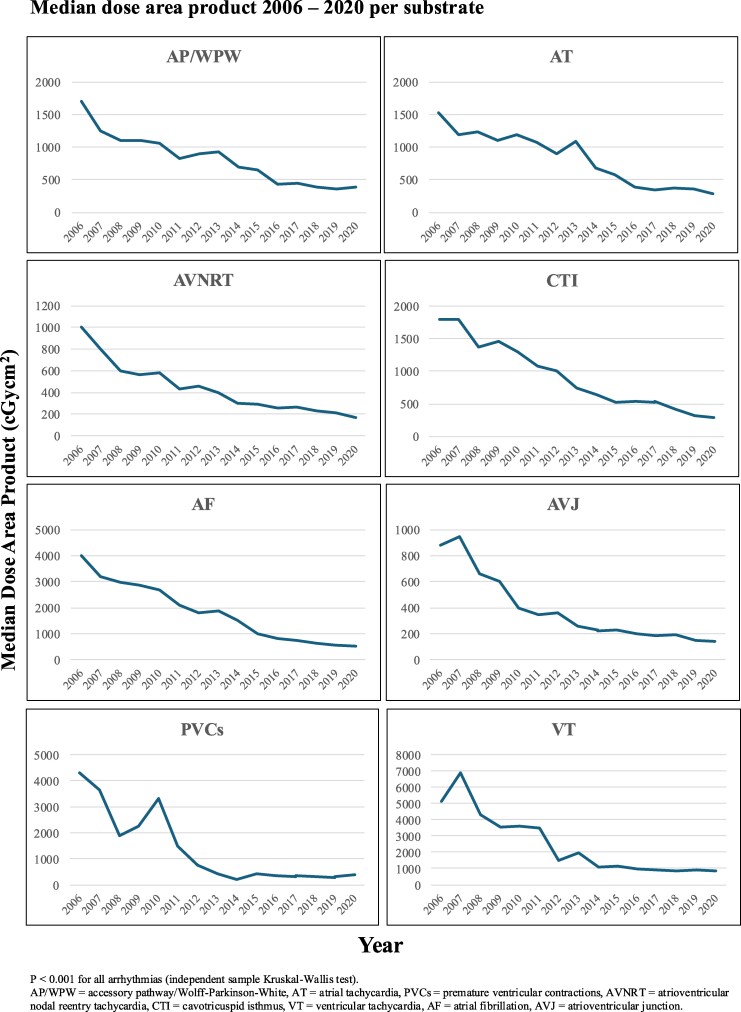
Median dose area product 2006–20 per substrate. *P* < 0.001 for all arrhythmias (independent sample Kruskal–Wallis test). AP/WPW, accessory pathway/Wolff–Parkinson–White; AT, atrial tachycardia; PVCs, premature ventricular contractions; AVNRT, atrioventricular nodal reentry tachycardia; CTI, cavotricuspid isthmus; VT, ventricular tachycardia; AF, atrial fibrillation; AVJ, atrioventricular junction.

Fluoroscopy time and median dose-area product (measured in cGycm^2^) showed a decreasing trend over time for all arrhythmias (*P* < 0.001 for all trends), see *Figure 2*. In ablation of AF, both fluoroscopy time and median dose area product showed significant reductions, with fluoroscopy time decreasing from 21 min (2006–15) to 12 min (2016–20; *P* < 0.001), and median dose area product from 1882 cGycm^2^ (2006–15) to 623 cGycm^2^ (2016–20; *P* < 0.001).

### Repeat ablation

**Figure 3:**
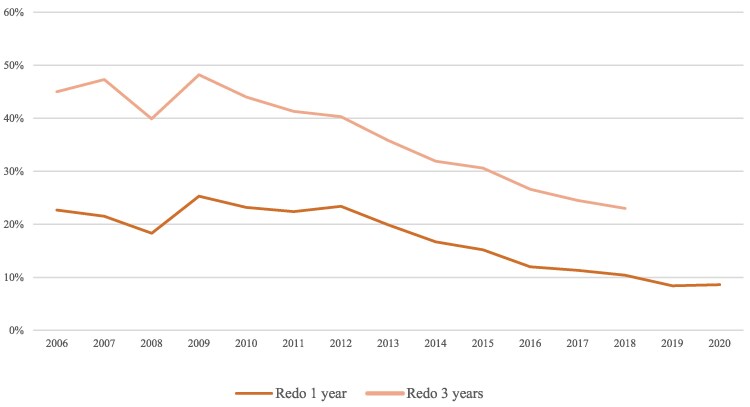
Rate of repeat ablation of *de novo* atrial fibrillation. Rate of repeat ablation in *de novo* atrial fibrillation ablations, deemed acute successful (by the operator at the end of the procedure).

**Figure 4:**
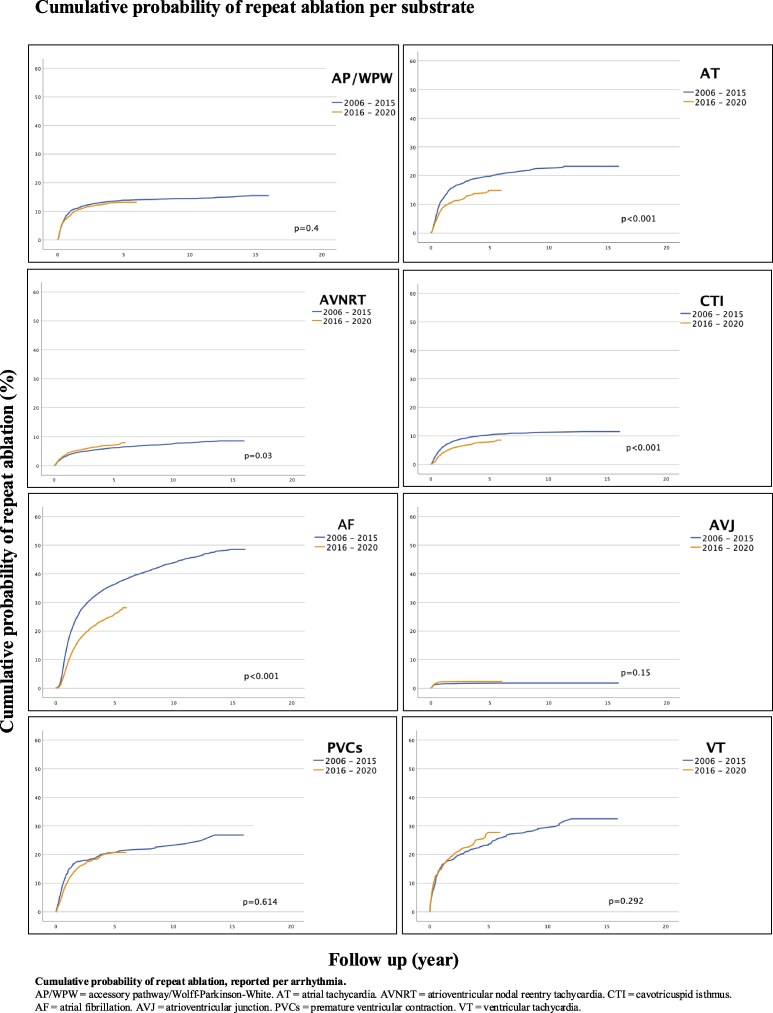
Cumulative probability of repeat ablation per substrate. Cumulative probability of repeat ablation, reported per arrhythmia. AP/WPW, accessory pathway/Wolff–Parkinson–White; AT, atrial tachycardia; AVNRT, atrioventricular nodal reentry tachycardia; CTI, cavotricuspid isthmus; AF, atrial fibrillation; AVJ, atrioventricular junction; PVCs, premature ventricular contraction; VT, ventricular tachycardia.

For AT, CTI, and AF, the cumulative probability of requiring a repeat ablation was significantly lower for procedures performed after January 2016, compared with procedures performed between 2006 and 2015 (*P* < 0.001 for all three types of arrhythmia). The risk of repeat ablation markedly decreased with each year for ablation of AF (see *Figure 3*). There was a non-significant trend (*P* = 0.292) suggesting an increased risk of repeat ablation of VT in procedures performed after January 2016, see *Figure 4*.

### Atrial fibrillation

**Figure 5:**
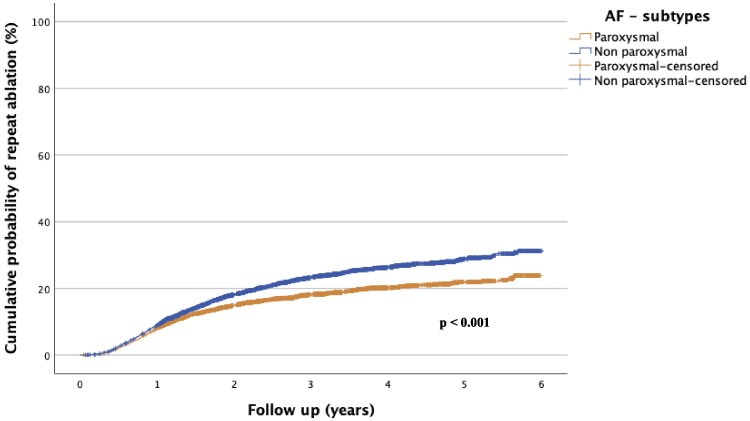
Cumulative probability of repeat ablation in paroxysmal vs. non-paroxysmal atrial fibrillation. Distribution among subtypes of atrial fibrillation 2016–20. Subtypes were not registered before 2016. AF, atrial fibrillation.

The increase in the annual number of total catheter ablations performed was primarily due to an increase in the number of AF procedures, see *Figure 1*. While RF was the dominant technique in AF ablation, there was an increase in the usage of the cryoballoon technique, from 12.9% for the period 2006–15 to 20.5% for the period 2016–20 (*P* < 0.001). The proportion of patients undergoing a repeat ablation of AF within 1 and 3 years, respectively, was shown to decrease when comparing the 2006–15 period (37 and 20%, respectively) to the 2016–20 period (20 and 10%, respectively), see *Figure 3*. There was no temporal difference in the distribution of subtypes of AF among patients undergoing ablations from 2016 to 2020, see *Table 2*. The risk of undergoing a repeat AF ablation was significantly higher if the index procedure was performed on non-paroxysmal AF (*P* < 0.001), see *Figure 5*.

**Table 2:** Distribution among subtypes of atrial fibrillation 2016–20

	2016 (*n* = 1297)	2017 (*n* = 1833)	2018 (*n* = 2423)	2019 (*n* = 2533)	2020 (*n* = 1540)
Paroxysmal AF	574 (44%)	819 (45%)	1056 (44%)	1196 (47%)	844 (55%)
Persistent AF	676 (52%)	942 (51%)	1242 (51%)	1233 (49%)	620 (40%)
Longstanding persistent AF	47 (4%)	72 (4%)	125 (5%)	104 (4%)	76 (5%)

Distribution among subtypes of atrial fibrillation 2016–20. Subtypes were not registered before 2016.

AF, atrial fibrillation.

### Complications

The total number of periprocedural complications was reported at 1.4% (470/34 166) for the period 2006–15, and 1.3% (338/26 269) for the period 2016–20 (*P* = 0.352). During the 2016–20 period, pericardial effusion/tamponade and bleeding at the local site were the most common reported complications, with an overall frequency of 0.4 and 0.3%, respectively. For the 2016–20 time period, the complication rate varied between arrhythmias, with the lowest frequency at 0.6% [10/1576, confidence interval (CI) 0.3–1.1] reported in catheter ablation of AVJ. Ablation of AF had a complication frequency of 1.5% (178/12 002, CI 1.3–1.7), constituting, but not limited to; tamponade (0.4%, 51/12 002), phrenic injury (0.1%, 12/12 002), stroke/transient ischemic attack (TIA) (0.3%, 35/12 002), and local bleeding/hematoma (0.2%, 30/12 002). In ablation of VT, complications were reported at 5.1% (47/926, CI 3.8–6.6) for the 2016–20 time period. The most frequent complication was cardiac tamponade at 1.5% (14/926).

## Discussion

In this nationwide multicentre study, based on a comprehensive healthcare registry with near-complete coverage, covering a 15-year period, a steady increase in the number of ablations performed annually was observed. The main driver behind the increase is the growing number of catheter ablations of AF. In patients with ablation performed after 2015, decreased risks of repeat ablation of AT, CTI, and AF were seen, with the most prominent decrease observed in ablations of AF. Although the risk of repeat ablation for AF decreases over time, there is no true plateau, meaning that even several years after the index procedure, some patients have recurring arrhythmia requiring repeat ablation.

### Population

The patients included in our study reflect a population treated in Sweden—a nation with a universal, single-payer, tax-funded healthcare system, ensuring equal access to healthcare for all residents regardless of socioeconomic status, and regardless of whether or not the individuals have private insurance. Age, gender distribution, and comorbidities in our study are similar to those in previous publications, and in line with the results of our study, a slight increase in median age for most ablations was observed from the year 2000 and forward in these previous publications.^5,10^ The indications for catheter ablation of arrhythmias have expanded during the studied years, and technical equipment has improved, gradually allowing physicians to treat older patients with more comorbidities. With expanded indications, an almost three-fold increase in the total number of procedures was observed from 2006 to 2019, with growing numbers each year. The slight decrease in the total number of ablations performed in 2020 reflects the result of the COVID-19 pandemic.

### Procedural data

Procedural times are generally shorter in the post-2016 time period for most arrhythmias, compared with the 2006–15 time period. The development of new techniques (e.g. pulsed-field ablation for ablation of AF) may decrease procedure times beyond the times reported in this study. Ablations of VT and PVCs break this trend, with a slight increase in procedural times, which is likely due to more complicated procedures being performed. This finding is consistent with a publication by Vaseghi *et al*., who investigated the outcomes of catheter ablation for VT in patients with dilated cardiomyopathy, arrhythmogenic right ventricular cardiomyopathy, myocarditis, hypertrophic cardiomyopathy, and sarcoidosis. Vaseghi *et al.*^11^ reported a slight difference in procedure times based on the underlying aetiology, with an overall mean procedure time of 295 ± 128 min.

### Fluoroscopy

We demonstrate a gradual decrease in the use of fluoroscopy over time, with the steepest decline seen during the 2006–15 time period. The current levels of fluoroscopy time and doses are low for all studied ablation types. The decrease could be due to the adoption and increased utilization of 3D-mapping systems, enabling ablations to be carried out with a minimal amount of radiation. The decrease was also observed in other publications reporting real-world data from large-scale centres.^2^ Despite more complex VT and AF ablations being performed, overall low levels of radiation exposure were observed in the last 5 years of the study. In ablations of VT, a slight increase was observed in median dose-area product, most likely again due to more complexity in ablation procedures being performed in recent years. With the stochastic effects of radiation, even with the now low levels of fluoroscopy, fluoroscopy may still pose a cumulative risk for both patients and staff. Efforts to further reduce radiation levels should be considered, in line with ALARA (as low as reasonably achievable), as well as efforts to develop techniques that allow zero fluoroscopy in order to protect the most vulnerable patients.^12–15^

### Repeat ablations

With years of development of technique and skills in catheter ablation, some improvement is expected in periprocedural data, which is evident in the significant decrease of repeat ablations after *de novo* procedures of AT, CTI, and AF shown in our study. However, in the remaining arrhythmia types, there is no clear difference between the 2006–15 and 2016–20 time periods. In ablation of AP/WPW, AVNRT, and AVJ, the development of new and improved techniques has likely had a lesser effect on the ablation procedures, illustrating that these procedures are still performed similarly to how they were done in the past. The recurrence and cumulative probability of PVCs and VT are more complex to interpret. While there is no statistically significant difference between the 2006–15 and 2016–20 time periods, there are still nominal differences in recurrences, with a discrete trend towards an increased need for repeat ablations in VT during the 2016–20 time period. It has previously been established that the success rate is lower in patients with structural heart disease compared with the success rate in patients with idiopathic VT.^16–18^ The increased trend in the need for repeat ablation procedures in VT observed in our study most likely reflects more complicated procedures being carried out, but could also be due to a lower threshold for readmission for re-ablation during recent years. Given the heterogeneity of patients undergoing ablations of VT, these procedures need to be studied on a larger scale, with the analyses stratified by subgroups to enable any drawing of conclusions regarding the long-term outcomes of ablations of VT and PVCs.

### Catheter ablation of atrial fibrillation

We observed an almost doubling of the number of AF ablations performed between the 2006–15 and 2016–20 time periods, and a six-fold increase in the number of AF ablations from 2006 to 2020. There was an overall decrease in procedure times, fluoroscopy time, and fluoroscopy dosage, despite a broadened indication for AF ablation. It is noteworthy that even though we observed an increase in one shot-devices (in this case, cryoballoon catheters) starting in 2016, which has been found to be associated with higher fluoroscopy usage,^19^ no similar upward trend of fluoroscopy usage was observed in catheter ablation of AF. The overall reductions seen in fluoroscopy time during AF ablations are most likely attributable to the significant reduction in fluoroscopy utilization during RF ablation. Overall, patients undergoing AF ablations seem to do increasingly better with fewer arrhythmia recurrences. In 2018, 20% of patients needed a repeat ablation within 3 years of the first ablation, while just a few years earlier, in 2009, almost 50% of patients needed a repeat ablation within 3 years of the first ablation. Recurrences of arrhythmia do not seem to reach a plateau, as with many other arrhythmias; instead, the risk of a repeat procedure persists even 15 years after the initial procedure, which is in line with previous publications.^10^ There is a clear difference in arrhythmia recurrence (measured as the need for repeat ablation) in patients who had procedures performed after 2016. Publications regarding comparisons among AF ablations performed per year are scarce, but are needed, since the results of our study point towards better outcomes of AF ablations during the 2016–20 time period compared with the 2006–15 time period. Even though the outcome of interest is AF relapse, this parameter is not recorded in the Registry. However, repeat ablation is reported and reflects the lower limit of AF relapse in the population (i.e. there will be individuals with AF relapse, in whom a repeat ablation is not performed). Moreover, it is unlikely that the indication for repeat ablation is stricter nowadays than previously. Therefore, the gradual decrease in the proportion of patients undergoing repeat ablation observed in our study is likely to reflect technical improvements, improved operator skills, and potentially improved patient selection. There was no difference in the distribution between subtypes of AF (paroxysmal or persistent), but a significantly lower risk of repeat ablation was observed if the initial ablation was performed on non-paroxysmal AF, in line with previous publications.^20,21^

### Complications

The frequency of periprocedural complications is low overall, <1.5%, with minimal difference between the 2006–15 and 2016–20 time periods. Certain arrhythmias (e.g. VT) are associated with a higher incidence of complications. While complications are likely underreported in the Registry (e.g. not covering complications that happen post-procedure), complications are presumably reported in a similar manner throughout the years, making it highly probable that the frequency of complications has not changed from 2006 to 2020, despite an increased total number of procedures and procedures being more complicated. While an increase in the number of procedures performed would most likely lead to a drop in the number of complications, this effect might be counterbalanced by more advanced procedures being carried out. Overall, compared with previous reports,^22–24^ the complication rates shown in our study are low. Haanschoten *et al*. examined outcomes in a cohort of 144 patients with post-infarction, drug-refractory VT undergoing catheter ablation. This high-risk population included nearly 50% of patients with a left ventricular ejection fraction below 30%. The Haanschoten *et al*.^16^ analysis showed an overall complication rate of 7.6%, which is similar to the 5.1% complication rate seen in our study, in a VT population that was overall healthier. This indicates that the major peri-procedural complications are likely adequately reported, although there may be underreporting of complications. Mol *et al.*^25^ performed a study on a European cohort of patients undergoing pulmonary vein isolation, demonstrating a complication incidence of 3.6%, showing that even in populations similar to the population in the present study (with similar health care systems), the frequency of complications reported in this study is likely too low.

### Future implications

Given the time span of this study, covering ablations from 2006 to 2020, pulsed field ablation (PFA) is not covered by this report since the technique was first described in human clinical study in 2021, by Verma *et al*.^26^ Pulsed field ablation has shown decreasing procedural times compared with cryoballoon technique with maintained safety profile,^27–29^ and in future reports similar to ours, one might expect decreased procedural times and fluoroscopy levels due to the use of PFA or other novel techniques. During the duration of the study, techniques used for ablation procedures have largely developed, as reported in a previous study by Boersma *et al.*^30^ and likely contribute largely to the improvement seen in outcomes, although not further mentioned or looked into in this study. Further development of known techniques and optimizing standard procedures (such as first-time AF ablation) with novel technologies would enhance the availability of catheter ablation procedures, which would address the anticipated increase in demand associated with the global ageing population.^31^ Future reports are likely to include older patients with more severe comorbidities (e.g. AF and heart failure), and it remains to be seen whether older patients with more severe comorbidities will affect trends in periprocedural data.

### Limitations

The Swedish Catheter Ablation Registry is a nationwide Swedish registry with high coverage, which offers detailed information about procedural characteristics. However, the information is limited in certain aspects—e.g. only complications occurring during the in-hospital stay of the procedure are reported. Recurrence of arrhythmias will happen in patients in whom further ablation procedures are, for whatever reason, deemed unwarranted. Hence, the reported rates of repeat catheter ablation procedures are to be considered the ‘floor’ of the true relapse rate of arrhythmias.

## Conclusions

In this nationwide Swedish study, we demonstrate a yearly increase in the number of ablations performed, driven primarily by a yearly increase in ablations of AF. Along with reduced procedural times and lower fluoroscopy levels, a reduction in the need for repeat ablations following index procedures for AF, AT, and CTI was observed, illustrating a substantial improvement in outcomes over the studied time period, particularly for AF ablations.

## Supplementary Material

oeaf142_Supplementary_Data

## Data Availability

The data that support the findings of this study are available from the Swedish Catheter Ablation Registry, but restrictions apply to their availability, which were used under license for the current study, and so are not publicly available. Data are, however, available from the authors upon reasonable request and with permission of the Swedish Catheter Ablation Registry.
