# Supplementary material for: Catheter ablation of arrhythmias: 15 years of development: data from the Swedish Catheter Ablation Registry
Source: Eur Heart J Open. 2025 Oct 23;5(6):oeaf142. doi: 10.1093/ehjopen/oeaf142 (PMC12625815; doi:10.1093/ehjopen/oeaf142)
Supplement: oeaf142_Supplementary_Data [file oeaf142_supplementary_data.docx]

**Supplementary material**

**Table 1**

**Coverage of each variable**

| **Variable** | **Percentage** |
| --- | --- |
| Ablation date | 100 % |
| Ablation year | 100 % |
| Date of birth | 99.7% |
| Ablation type | 100% |
| Age | 100% |
| Gender | 100% |
| Length | 24% |
| Weight | 67.4% |
| Subtype of AF | 51.5% |
| IHD | 71.9% |
| DCM | 34.7% |
| HCM | 34.5% |
| ARVC | 60% |
| Procedure time | 97.8% |
| Fluoroscopy time | 97.6% |
| Median Dose Area product | 97.1% |
| RF ablation | 97% |
| RF energy | 55.9% |
| RF time | 77.7% |
| Cryo ablation | 97% |
| Cryo time | 97.1% |
| Acute success | 92% |
| Adverse event | 100% |
| Date first redo | 100% |
| Redo procedure | 100% |
| Future redo | 100% |
| Redo after 1 year | 100% |
| Redo after 3 years | 100% |
| Death | 100% |
| Follow up | 100% |
